# Neurological Soft Signs in the Clinical Course of Schizophrenia: Results of a Meta-Analysis

**DOI:** 10.3389/fpsyt.2014.00185

**Published:** 2014-12-23

**Authors:** Silke Bachmann, Christina Degen, Franz Josef Geider, Johannes Schröder

**Affiliations:** ^1^Clienia Littenheid AG, Littenheid, Switzerland; ^2^Department of Psychiatry, Psychotherapy, and Psychosomatics, University of Halle, Halle, Germany; ^3^Section of Geriatric Psychiatry, Institute of Gerontology, University of Heidelberg, Heidelberg, Germany

**Keywords:** NSS, schizophrenia, chronicity, course, outcome

## Abstract

Neurological soft signs (NSS) comprise subtle deficits in sensory integration, motor coordination, and sequencing of complex motor acts, which are typically observed in the majority of schizophrenia patients, including chronic cases and neuroleptic-naïve first-episode patients. However, recent studies clearly demonstrate that NSS are not a static feature of schizophrenia but vary in the clinical course of the disorder. This effect was investigated in a meta-analysis based on 17 longitudinal studies published between 1992 and 2012. Studies included between 10 and 93 patients with schizophrenia spectrum disorders (total number 787) with follow-up periods between 2 and 208 weeks. Beside the Neurological Examination Scale, the Cambridge Neurological Inventory and the Heidelberg NSS Scale were used to assess NSS. All but three studies found NSS to decrease in parallel with remission of psychopathological symptoms. This effect was more pronounced in patients with a remitting compared to a non-remitting, chronic course (Cohen’s *d* 0.81 vs. 0.15) and was significantly correlated with length of the follow-up period (*r* = −0.64) but not with age (*r* = 0.28). NSS scores did not decrease to the level typically observed in healthy controls. From a clinical perspective, NSS may therefore be used to identify subjects at risk to develop schizophrenia and to monitor disease progression.

## Introduction

Neurological soft signs (NSS) refer to subtle neurological abnormalities comprising deficits in sensory integration, motor coordination, and sequencing of complex motor acts (1–3). It is generally accepted that NSS are more prevalent in schizophrenia patients compared to healthy subjects. They have consistently been demonstrated in neuroleptic-naïve first-episode patients, i.e., prior to medication exposure, supporting the assumption that NSS constitute an intrinsic feature of schizophrenia. This notion is underlined by increased NSS scores in high-risk subjects, such as relatives of schizophrenic patients, or in the unaffected co-twins of monozygotic twin-pairs discordant for schizophrenia (4–6).

Already in 1980, Torrey found NSS to be associated with more chronic and severe forms of the illness (7). Along these lines, Manschreck and Ames (1) reported significant correlations between NSS and psychopathological symptoms in a cross-sectional study. These findings led to the hypothesis that NSS are not a static feature of schizophrenia but vary in the clinical course of the disorder, which was investigated in the late 1980s and early 1990s by the Heidelberg group (3, 8). Patients with an acute exacerbation of schizophrenia were investigated on admission, the seventh day of treatment, and following remission of acute symptoms, prior to discharge. Results clearly demonstrated a decrease of NSS scores with remission of psychopathological symptoms. Similarly, the parallel decrease of NSS and psychopathological symptoms during neuroleptic treatment was confirmed in drug-naïve first-episode (FE) patients (9) and during the long-term course up to 4 years (10–12). When contrasted with healthy controls, NSS scores obtained in schizophrenia patients remained significantly higher during follow-up indicating that NSS comprise both state- and trait-features.

These findings are pivotal to our concept of NSS and indicate that cerebral sites important for motor and sensory functions are directly involved in schizophrenia. From a clinical standpoint, NSS may be used to monitor the disease process or to identify subjects with an increased liability toward schizophrenia. However, these conclusions are based on the above observation of the remission of NSS with psychopathological symptoms during the clinical course. In the present study, we therefore analyzed the development of NSS in the course of schizophrenia as it was described in longitudinal studies. We hypothesized NSS scores to decrease with remission of psychopathological symptoms and expected this effect to be more pronounced in patients with a more favorable compared to a chronic, non-remitting course of disorder.

## Methods

Studies on the longitudinal course of NSS in schizophrenia were identified by conventional bibliographic search in particular of the reference list of previous papers (13) and by a *pubmed* search performed in August 2014 using the keywords: “schizophrenia,” “NSS,” “course,” “follow-up,” “chronicity.” These procedures yielded 16 relevant publications, which reported the data necessary to calculate effect sizes (Cohen’s *d*). An additional data set was contributed on request (14). Studies were published between 1992 and 2012; they differed with respect to the clinical settings and the rating instruments used (Table 1). Eleven studies assessed NSS with the Neurological Examination Scale (NES) and four with the Heidelberg scale (HD). One study used the Cambridge Neurological Inventory [CNI (15)]. Two studies examined adolescents (16, 17). The latter results will be discussed separately, since the motor system has not entirely maturated at this age. Separate effect sizes were calculated for NSS and psychopathology before estimating mean effect sizes for patients with remitting and chronic courses.

**Table 1:** **Overview of studies**.

Study	Follow-up (weeks)	Scale	N	Diagnostic groups	Mean age (SD)	Medication	NSS t1	NSS t2	Effect size	Psy. path t1	Psy. path t2	Effect size 2
Bachmann et al. (10)	56	HD, PANSS	39	FE (DSM-IV)	27 (7.7)	Clin. Needs	15.7 (7.1)	10.1 (7.9)	0.75	52.4 (25.6)	52 (12.4)	0.02
			22	HC	28 (3.8)		4.8 (3.3)	4.6 (3.9)	0.06	*	*	
			18	NR (DSM-IV)	*		13.8 (7.2)	13.5 (8.7)	0.04	52.6 (14.9)	63.9 (32.6)	−0.48
			21	R (DSM-IV)	*		17.3 (6.8)	7.2 (5.8)	1.60	51.4 (10)	42.6 (10.8)	0.85
Boks et al. (18)	104	NES, PANSS	29	FE (DSM-IV)	26.9 (6.3)		7.5 (7.1)	8.9 (5.5)	−0.22	*	*	*
Buchanan et al. (19)	10	NES, BPRS	16	CH (DSM-III)	34.1 (6.8)	Clozapine	16.2 (5.9)	16.8 (8.2)	−0.09	11.4 (5.8)	9.4 (5.2)	0.34
	10		15	CH (DSM-III)	34.6 (9.1)	Haloperidol	14.5 (6.3)	15.1 (7.5)	−0.09	12.6 (5.3)	12.0 (5.1)	0.12
Chen et al. (20)	156	motor CNI	93	FE (DSM-IV)	31.2 (9.6)	Haloperidol imitially	1.87 (2)	1.45 (2.2)	0.2	*	*	*
			68	HC	32 (8.4)		*	*		*	*	*
Cuesta et al. (21)	26	NES	77	FE (DSM-IV)	30.1 (10)	Risperidone or olanzapine	17.1 (9.4)	9.9 (6.8)	0.89	10.1 (3.5)^a^	1.6 (2.1)	3.04
		SAPS, SANS			30.6 (6.2)		*	*		8 (5.9)_b_	4.7 (4.9)	0.61
Emsley et al. (14)	52	NES	15	FE (DSM-IV)	28.1 (8.5)		6.2 (3.57)	5.1 (4.0)	0.3	*	*	*
Mangot and Sawant (22)	52	NES	40	FE (ICD-10)	35.5 (11.9)		8.5 (7.1)	3.3 (4.1)	0.93	*	*	*
Mayoral et al. (16)	104	NES	29	FE (DSM-IV)	15.7 (1.6)		23.2 (9.1)	19.2 (9.9)	0.42	*	*	*
			22	HC	15.2 (1.6)		12.2 (6.7)	9.7 (5.2)	0.42	*	*	*
Mayoral et al. (17)	104	NES, PANSS	69	FE (DSM-IV)	15.5 (1.8)		25.2 (9.6)	19.9 (8.1)	0.6	66.2 (17.9)	58 (23.3)	0.40
			80	HC	15.2 (1.9)		11.1 (7.2)	9.2 (5.5)	0.3	*	*	*
Mittal et al. (23)	6	Quitkin, BPRS	19	SCHIZ (DSM-III-R)	36.3 (5.4)	Haloperidol	6.3 (0.9)	5.3 (0.8)	1.18	34.3 (2.1)	22.4 (2.2)	5.53
Prikryl et al. (11)	52	NES, PANSS	92	FE (ICD-10)	25.3 (5.5)		*	*		*	*	
			20	NR	*		6.5 (4.1)	4.2 (4.1)	0.56	88.4 (19.9)	84.7 (21.6)	0.18
			72	R	*		5.3 (5.9)	2.7 (3.4)	0.56	97.6 (22.5)	43.8 (11.1)	3.20
Prikryl et al. (12)	208	NES	68	FE (ICD-10)	22.5 (5)		6.3 (5.1)	6.8 (6.6)	−0.09	97 (23.2)	51.5 (19)	2.16
			29	NR	*		6.6 (4.8)	10.1 (7.6)	−0.56	*	*	*
			39	R	*		6 (5.4)	4.4 (4.5)	0.32	*	*	*
Schröder et al. (3)	variable	HD, BPRS	27	CH (DSM-III)	36 (12.1)	Clin. needs	27.8 (9.2)	22.1 (7.1)	0.7	*	*	*
			23	R	28.9 (8.9)		23.5 (8.3)	13 (4.7)	1.61	*	*	*
Schröder et al. (8)	variable	HD, BPRS	32	CH and R (DSM-III)	32 (9)	Clin. needs	21.3 (8.3)	11.5 (5.7)	1.4	46.1 (7.3)	32.1 (5.7)	2.15
Schröder et al. (9)	4	HD, BPRS	15	FE (DSM-III-R)	29.2 (9.4)	Benperidol	16.2 (7.5)	10 (4.7)	1.02	48.1 (6.6)	35.7 (7.7)	1.75
			8	R			15.5 (7.2)	9.3 (4.6)	1.05	49.4 (7.8)	32.5 (7.2)	2.25
			7	NR			17.0 (8.5)	10.9 (5.0)	0.9	46.7 (5.2)	39.3 (7.4)	1.18
Sevincok and Topaloglu (24)	2	NES, PANSS	10	CH (DSM-IV)	24.5 (*)	Olanzapine	19.1 (13.2)	14.7 (12.5)	0.34	78.8 (19.9)	58.0 (13.1)	1.26
Whitty et al. (25)	26	NES, PANSS	79	FE (DSM-IV)	23.4 (*)		15.6 (9.7)	12.5 (7.3)	0.36	83.3 (20.1)	58.4 (15.1)	1.41

HD, Heidelberg NSS Scale; PANSS, Positive and Negative Syndrome Scale; NES, Neurological Evaluation Scale; BPRS, Brief Psychiatric Rating Scale; CNI, Cambridge Neurological Inventory; SAPS, Scale for the assessment of positive symptoms; SANS, Scale for the assessment of negative symptoms; FE, first-episode psychosis; HC, healthy controls; NR, non-remitters; R, remitters; CH, chronic schizophrenics; SCHIZ, schizophrenia patients; ^a^, SAPS, ^b^, SANS, *, missing values.

## Results

Included studies are summarized in Table 1. The number of patients per study ranged between 10 and 93 (total number 787); 12 studies solely included FE patients, 2 studies focused on adolescents. Patients’ clinical course was characterized as either remitting or non-remitting/chronic in eight studies; in one study only the diagnosis “schizophrenia” was conveyed. Follow-up periods ranged between 2 weeks and 4 years. In nine studies, the examination commenced during the acute psychotic episodes; in four of those a reexamining after remission of acute symptoms, prior to discharge, took place. Standardized neuroleptic treatment was prescribed in five studies, namely high potency butyrophenones (haloperidol or benperidol), risperidone, or olanzapine.

All but three studies described a decrease of NSS in the clinical course. Eleven studies included an assessment of the clinical course. Patients with remitting symptoms showed a steeper decrease of NSS scores (mean effect size: Cohen’s *d* 0.81) than patients with an unfavorable or chronic course (mean effect size: Cohen’s *d* 0.15), who even exhibited an increase in scores. Overall, effect sizes (Cohen’s *d*) ranged between −0.56 (patients with an unfavorable course) and 1.61 (patients with a remitting course treated for an acute episode) with an overall mean effect size of Cohen’s *d* 0.53. Ratings of psychopathological symptoms paralleled the development of NSS scores but were only reported in 11 of the 17 studies. Moreover, data on the distinction between negative and positive symptom scores were provided by five studies only (12, 17, 21, 23, 24). In FE patients, as a group, a moderate effect size was present (Cohen’s *d* 0.42). Similar effect sizes (Cohen’s *d*: 0.42–0.60) were reported in adolescent patients with FE psychosis. Effect sizes were significantly (*p* < 0.05) correlated with length of the follow-up period (*r* = −0.64, *p* = 0.001) but not with age (*r* = 0.28).

## Discussion

The present meta-analysis revealed two main findings: (i) a confirmation that NSS scores decrease in the clinical course of schizophrenia with remission of psychopathological symptoms; and (ii) an indication that this effect is more pronounced in patients with a remitting course than in those with non-remitting schizophrenia.

Despite numerous methodological differences, all but three studies found decreasing NSS scores during the clinical course with remission of acute schizophrenia symptoms. Boks et al. (18) reported NSS to increase in a group of 29 FE patients, who were investigated 2 years apart (Cohen’s *d* −0.22). They assigned this effect to a subgroup of patients with a more severe form of the disorder or a regression to the mean and stressed the importance of the relatively small sample size when interpreting their findings. A marginal increase of NSS scores (Cohen’s *d* −0.09) in patients with chronic schizophrenia was also reported by Buchanan and colleagues (19) in 31 patients who received haloperidol or clozapine for 10 weeks. Jahn et al. (26) identified increasing NSS scores only in a small subgroup of patients in whom symptoms deteriorated. Their study could not be included in our meta-analysis, because only median values had been documented. Prikryl et al. (12) systematically followed FE patients for 4 years and found NSS to marginally increase (Cohen’s *d* −0.09) when the whole group of 68 patients was considered. This effect, however, was caused by a subgroup of patients developing chronic schizophrenia (Cohen’s *d* −0.56) while NSS in those with a remitting course decreased (Cohen’s *d* 0.32). A similar build-up of NSS scores has been described by Chen et al. (27) in 43 patients with chronic schizophrenia over the course of 3 years. Their study was not included here, because total NSS scores had not been provided. Taken together, effect sizes for the decrease of NSS scores ranged from Cohen’s *d* 0.04 to Cohen’s *d* 1.61 with an average of Cohen’s *d* 0.64. The smallest effects were seen in patients with non-remitting schizophrenia (mean effect size for non-remitting patients: Cohen’s *d* 0.15) while more pronounced effect sizes were observed in patients with a remitting course (mean effect size for remitting patients: Cohen’s *d* 0.81). However, only eight studies assessed the clinical course while nine just characterized patients as FE or solely documented DSM diagnoses.

A most interesting question arises from the finding that the decrease of NSS and the decrease of symptoms parallel each other. Unfortunately, this relationship could not be analyzed any further by meta-analytic tools. Both NSS and psychopathology had been assessed with several different instruments, and symptom scores were not provided in several publications.

The effect sizes reported in FE studies appeared to be lower than those found in patients with a remitting and even those with a chronic course. However, only four of the available FE studies drew a distinction between remitting and non-remitting patients. In addition, the exact timing of the first examination after study intake has to be taken into account. While Prikryl and colleagues (11, 12) and Schröder et al. (9) examined NSS at or shortly after admission, i.e., in an acute psychotic state – Bachmann et al. (10) conducted the first NSS examination after clinical stabilization, before discharge. This may well have had an impact on the findings since NSS scores typically show a significant decrease with remission of the acute symptoms (3, 8). The important meta-analysis of cross-sectional NSS studies by Chan et al. (28) found effect sizes of patients vs. controls comparisons to be moderated by duration of illness. One may hypothesize that NSS continue to improve or worsen during the course following the first manifestation of the disease; a hypothesis, which conforms to the above cited studies, namely the differences, which emerged between patients with a remitting vs. a non-remitting, chronic course.

The decrease of NSS with clinical stabilization during the course was more pronounced in patients with a favorable than with a non-remitting course (3, 9, 10, 11, and 12); therefore, NSS scores could be identified as course predictors. However, even after remission of the acute illness, NSS scores remained significantly higher than in healthy controls. These findings demonstrate that NSS in schizophrenia are both trait- and state-related. NSS persistence and deterioration in patients with a chronic course of the disorder clearly point to a progression of corresponding cerebral changes as demonstrated in a recent study from the Heidelberg group (29). From a clinical perspective, NSS may therefore be used to identify subjects at risk to develop chronic schizophrenia. It is well known, that total NSS scores in schizophrenia are mainly due to motor and sensory subscores. Unfortunately, only 7 of 17 studies (10–12, 16, 17, 21, 25) reported subscores; therefore, a more detailed meta-analysis could not be performed.

A decrease of NSS scores with clinical stabilization was also reported in adolescent patients with FE psychosis (16, 17), i.e., during an age in which the motor and sensory systems are still not entirely maturated. Both studies of the Mayoral group also found a decrease of NSS in the control group over the 2-year-study period and a higher score level in patients on comparison. Authors attributed their findings to an overall developmental delay caused by rather than a direct consequence of the disease.

Potentially confounding variables include different NSS rating scales and study designs. Although different, the various NSS scales are comparable with regard to the majority of subscales and sensitivity (27); moreover, the potential impact of the respective psychometric differences is at least partially addressed by the longitudinal designs of the studies. Neuroleptic treatment was standardized in 6 of the included 17 studies only. However, the vast majority of studies discussed here report decreasing NSS in patients treated with any neuroleptic compound. Furthermore, it is known that NSS are not caused by neuroleptic medication (30, 31). In our study, a negative correlation between effect sizes and length of follow-up period was identified. It is plausible that clinical stabilization occurs in the beginning of the follow-up interval leaving mostly non-remitting patients. As a result, effect sizes of NSS decrease with length of inspected interval.

Along with this, Buchanan et al. (19) did not find meaningful or even significant differences in chronic patients receiving either clozapine or haloperidol while Schröder et al. (9) demonstrated a decrease of NSS in FE patients who received a conventional neuroleptic. As discussed above, the timing of the first examination may even have great importance, in particular as the exact starting points were not defined in a number of studies. The decrease of NSS was correlated with age, although this did not reach statistical significance. The meta-analysis of cross-sectional studies (28) did not find NSS difference scores between patients and healthy controls to be moderated by age when only studies with the NES were entered into the meta-analysis. Although both findings correspond with respect to the direction of change, the impact of age and other potential moderators as sex and education, or cognitive reserve (Urbanowitsch et al., submitted) on NSS needs to be further analyzed. Against the background of a relatively small number of studies included here, the respective methodological questions call for a large multicenter longitudinal study in FE patients. Such an endeavor would not only be suitable to dissect the decrease of NSS in the clinical course confirmed in this meta-analysis but could also serve to define the state and trait characteristics of NSS more precisely. The potential impact of motor development could be addressed in adolescent patients; moreover, neuroimaging studies could be designed to better understand the role of motor and sensory cortical sites in schizophrenia.

From a clinical standpoint, the decrease of NSS with clinical stabilization may be used to monitor disease progression or to identify subjects with an increased liability toward schizophrenia in general and more chronic, unfavorable courses in particular. As a phenomenon, NSS point at the involvement of motor and sensory cerebral sites in the disorder. Since NSS correspond to impaired motor and sensory functions like motor coordination, they can be used to further develop and optimize physical training programs, which are already part of the routine therapeutic repertoire.

## Conflict of Interest Statement

The authors declare that the research was conducted in the absence of any commercial or financial relationships that could be construed as a potential conflict of interest.
